# Analysis of Deformation and Properties of Composite Melon Petals via Vibration Pretreatment—Microwave Compound Curing

**DOI:** 10.3390/polym15234541

**Published:** 2023-11-26

**Authors:** Chenglong Guan, Tongming Chi, Lihua Zhan, Junhao Chen, Bing Wang, Liping Xie, Shuncong Zhong

**Affiliations:** 1 Fujian Provincial Key Laboratory of Terahertz Functional Devices and Intelligent Sensing, School of Mechanical Engineering and Automation, Fuzhou University, Fuzhou 350108, China; guancl@fzu.edu.cn (C.G.); 230227069@fzu.edu.cn (T.C.); 230220068@fzu.edu.cn (J.C.); lpxie@fzu.edu.cn (L.X.); sczhong@fzu.edu.cn (S.Z.); 2 College of Mechanical and Electrical Engineering, Central South University, Changsha 410083, China; 3 Light Alloys Research Institute, Central South University, Changsha 410083, China

**Keywords:** cryogenic composite tank, melon petal, vibration pretreatment, microwave curing, deformation deviation, permeation rate

## Abstract

The transition of large-scale cryogenic propellant tanks from metal to composite materials is the main trend in the global aerospace industry. Aiming to address the challenges of achieving the manufacturing of integrated and cost-effective manufacturing of aerospace cryogenic composite tanks that cannot be realized through the conventional autoclave process, and those of existing out-of-autoclave processes that are unable to effectively suppress defects under low-pressure conditions, a vibration pretreatment was innovatively introduced into the microwave curing process of composite materials in this study. Based on a systematic analysis of the inhibitory mechanisms of vibration pretreatment on void formation and the uniform heating mechanisms of microwaves in composite materials, the experimental results showed that the compound curing process enabled the production of components with complex structural features under low-pressure conditions while achieving equivalent surface precision and comprehensive properties, including porosity, interlaminar shear strength, and cryogenic permeation resistance, as those obtained through the standard 0.6 MPa autoclave process. This holds great promise for the application of out-of-autoclave processes in the manufacturing of large-scale aerospace cryogenic composite tanks.

## 1. Introduction

The relentless pursuit of structural lightweighting and maximal efficiency stands as a perpetual theme in the advancement of aerospace vehicles. Within this context, the cryogenic tank emerges as a pivotal structural element within aerospace carriers, serving the dual role of cryogenic propellant storage and being a load-bearing structure. The imperative for lightweight design and manufacturing in this component assumes paramount importance, particularly for heavyweight launch vehicles [1]. The cryogenic tank’s mass constitutes over 60% of the carrier structure’s dry weight, rendering the reduction of its weight a definitive factor governing overall structural lightweighting [2,3,4]. Comprehensive investigations conducted by Boeing Company have substantiated that composite tanks can realize weight reductions ranging from 20% to 40% in comparison to metal tanks, concomitantly slashing manufacturing costs by more than 25% [5,6]. Consequently, ultra-large composite tanks have emerged as pivotal components which are poised to propel advancements in the launch efficiency of aerospace vehicles and reduce the cost of the aerospace industry, underpinning the next generation of launch vehicles, deep-space exploration missions, and in-orbit space stations [7].

Research institutions, led by the National Aeronautics and Space Administration (NASA), embarked on the development of liner-less composite cryogenic tanks as early as the 1980s and 1990s. Over the years, they have achieved a series of significant advancements. Representative examples encompass the Φ2.4 m liquid hydrogen tank realized during NASA’s DC-XA program in 1996 [8,9], the Φ3 m liquid hydrogen tank developed by Lockheed Martin Company (LMT) in support of the X-33 program during the late 1990s [8], the Φ2.4 m and Φ5.5 m composite tanks produced for NASA by Boeing in 2011, employing an innovative segmented tooling approach [10], and the Φ3.35 m liquid oxygen tank manufactured by the China Academy of Launch Vehicle Technology (CALT) in 2021 under the National Key Research and Development Program [11], among others.

Due to the non-weldable characteristics of composite materials and the stringent cryogenic media impermeability requirements for tanks [12], the prevailing technology that is currently embraced involves integrating fiber winding and automated fiber placement with autoclave processes to mitigate internal porosity in the components. However, with the burgeoning demand for an enhanced rocket launch efficiency and cost reduction necessitating the continual enlargement of tank diameters, the conventional heating approach based on convection and conduction within autoclave processes inevitably results in pronounced temperature disparities between the windward and leeward ends of the tank [13,14,15]. Furthermore, the uneven heating of the mold further exacerbates temperature gradients along the thickness of the tank [16]. Such spatial temperature heterogeneity can engender uneven resin flow and asynchronous curing inside the component, which not only leads to the occurrence of defects such as voids and delamination, but also induces residual stresses and curing deformations. On the other hand, the hermetic constraints of the autoclave significantly restrict the maximum diameter of integrated composite tanks, and extended manufacturing cycles and heightened energy consumption also present formidable challenges within the autoclave process [17,18]. Consequently, NASA has underscored in multiple reports the imperative need for out-of-autoclave (OOA) processes to facilitate the production of ultra-large-diameter composite tanks [6,8,19].

Microwave curing, an emerging out-of-autoclave molding process, has gained substantial attention in recent years within the field of composite curing due to its remarkable advantages, including rapidity, energy efficiency, and heightened productivity [20,21]. In contrast to conventional autoclave processes, microwave curing is not encumbered by pressure-vessel size constraints, and it boasts a heating rate exceeding 15 °C/min, significantly elevating production efficiency and cost-effectiveness. Notably, its distinctive heating mechanism circumvents the challenges associated with large and intricate structural features, ensuring uniform temperature distribution [22]. This effectively resolves the technical hurdles encountered in the autoclave curing of sizable aerospace composite tanks [23,24]. Nonetheless, the high-quality manufacturing of aerospace composite tanks hinges heavily on the curing pressure, while standard microwave heating systems often struggle to maintain continuous operation under high-pressure conditions. Atmospheric or low-pressure curing conditions not only fail to effectively inhibit the formation of defects such as voids and delamination, but also significantly compromise the macro-/micro-mechanical properties of components. Furthermore, internal defects can establish pathways within the tank structure, potentially leading to permeation and tank failure when storing cryogenic propellants [12,25]. To address these challenges, extensive research has been conducted, with a predominant approach being the integration of microwave technology with autoclave systems to provide the necessary pressure for composite forming [26,27,28,29]. However, this approach does not fundamentally overcome the limitations of autoclaves in terms of component size and pressure dependence, and further escalates energy consumption and manufacturing expenses, rendering it unsuitable for the out-of-autoclave formation of large-scale cryogenic composite tanks.

Hence, by building upon prior research findings that introduced vibration treatment into the microwave curing process of composite materials, effectively mitigating internal porosity issues and enhancing the molding quality [30,31,32], this study pioneers the application of this innovative technology in the production of composite cryogenic tanks. A systematic analysis was conducted to elucidate the inhibitory mechanisms of vibration treatment on porosity formation and the uniform heating mechanisms of microwaves on composite materials. Utilizing composite melon petals that mimic typical tank structural features as the research subject, in conjunction with the self-developed experimental vibration equipment and microwave heating device, a process involving vibration pretreatment followed by microwave curing for out-of-autoclave molding was executed, and the standard autoclave process was chosen as the experimental control group. Deformation deviations after molding were analyzed by scanning the surfaces of the melon petals using a three-dimensional scanner. The porosity and microstructure within the complex structural features of the components were investigated through Optical Digital Microscopy (ODM). The impacts of different processes on the mechanical properties of the petals were assessed via short-beam three-point bending tests and further analysis of the sample fracture surfaces was conducted using Scanning Electron Microscopy (SEM). Simultaneously, with a focus on the specific evaluation criteria for aerospace cryogenic tanks, particularly permeation resistance, a test apparatus was established to replicate the actual service environment of the tanks, and a series of tests was conducted to verify the engineering application prospects of the compound curing process in the out-of-autoclave manufacturing of aerospace composite tanks.

## 2. Theories in Vibration Pretreatment–Microwave Curing Compound Process

### 2.1. Mechanism of Void Reduction by Vibration Treatment

The evolution of voids within composite components during the curing process is influenced by a multitude of factors. Within composite laminates, the formation of voids is primarily driven by the diffusion of mixed air and impurity molecules as the curing temperature rises. The coalescence and subsequent re-dissolution of adjacent voids can significantly contribute to the expansion a void. In contrast, alterations in the temperature and pressure throughout the curing process may enhance the solubility of the resin, potentially inhibiting void growth and, in some cases, leading to void collapse. Consequently, a comprehensive analysis of the stability equation for voids could elucidate the principles by which vibration pretreatment effectively mitigates void formation.

**Figure 1:**
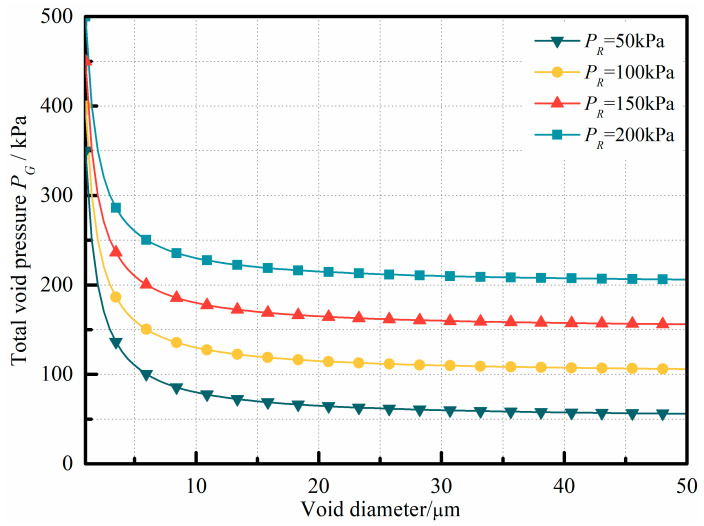
Relationship curves between void pressure and diameter in a typical resin system.

When the internal pressure within a void is balanced with the combined pressure exerted by the surrounding epoxy resin and its surface tension, the void can maintain a stable configuration. The equilibrium equation is represented in Equation (1):

(1) $$P_{G}-P_{R}=\frac{\gamma_{LV}}{m_{LV}}$$

where $P_{G}$ and $P_{R}$ represent the void and resin pressures, respectively. $\gamma_{LV}$ is the resin–void surface tension, and $m_{LV}$ is the ratio of the void volume to its surface area. In this study, under the condition of a lower resin viscosity caused by the increased curing temperature, the resin-induced pressure comprises three parts:

(2) $$P_{R}=\rho gh+P_{E}+P_{Vir}$$

where ρgh represents the hydrostatic pressure of resin, $P_{E}$ is the external air pressure, and $P_{Vir}$ represents the force provided by the vibration treatment. For analytical convenience, assuming that the voids are uniform spheres, Equation (1) can be rewritten as follows:

(3) $$P_{G}=P_{R}+\frac{6\gamma_{LV}}{d_{LV}}$$

where $d_{LV}$ represents the void diameter. Choosing the resin–void surface tension $\gamma_{LV}=0.05\;N/m$, correlation curves of the internal void pressure versus the void diameter, with $P_{R}$ as the controlling parameter, can be plotted, as shown in Figure 1.

Figure 1 illustrates the equilibrium curves representing void behavior at varying liquid resin pressures. For all curves in the figure, voids situated above the curve will continue to expand, while those below it will gradually collapse. Referring to Equation (2), the introduction of vibration amplifies the pressure term within the resin, thus increasing its value. As demonstrated by Equation (2), the introduction of vibration augments the pressure term $P_{Vir}$ within the resin pressure term $P_{R}$, resulting in an increase in its value. In conjunction with Figure 1, it becomes evident that, under constant void internal pressure $P_{G}$, as the resin pressure term $P_{R}$ increases, a larger void diameter is necessary for stability within the resin. For instance, when the void internal pressure is set at $P_{G}=200\;\mathrm{kPa}$, if the resin pressure $P_{R}$ reaches or exceeds 200 kPa, theoretically, the void diameter required for stable existence within the resin will tend towards infinity. In other words, in this case, there will be no voids that can stably exist within the laminate, and all voids will collapse under the action of the external resin pressure. Consequently, during vibration pretreatment, if the mechanical vibration can supply a sufficiently high field strength and energy input, voids within the liquid resin will be unable to maintain their equilibrium and will inevitably collapse.

On the other hand, following the law of energy conservation, the energy introduced during vibration pretreatment will be converted into the material’s internal energy, leading to an overall increase in the temperature of the resin [33]. This increase in temperature, as described by Equation (4), subsequently causes a reduction in the resin’s viscosity:

(4) $$\eta =\eta_{0}\exp\left(\frac{Q}{RT}\right)$$

where $\eta_{0}$ is the initial viscosity of the resin, Q denotes the energy absorbed by the resin matrix, R is the molar constant of the gas, and T is the ambient temperature. Therefore, in conjunction with Stokes’ law (Equation (5)) and Archimedes’ principle (Equation (6)), the decrease in the resin’s viscosity, as described earlier, facilitates the more rapid ascent of voids within the resin, and the voids then move toward the surface of the component under the influence of the vibration energy, as depicted in Equation (7). Supported by the vacuum system, these voids are promptly expelled from the component. In summary, the mechanism through which vibration pretreatment mitigates defects within composite components can be characterized as the collapse of voids due to the influence of vibrational energy and their subsequent removal aided by the vacuum system.

(5) $$F=\frac{4\pi }{3}r_{V}^{3}\rho_{R}g$$

(6) $$F=6\pi r_{V}\eta \nu$$

(7) $$\nu =\frac{2}{9}\frac{\rho_{R}r_{V}^{2}}{\eta g}=\frac{1}{18}\frac{\rho_{R}d_{V}^{2}}{\eta g}$$

where F represents the void buoyancy, $r_{V}$ is the void radius, $\rho_{R}$ denotes the resin density, g is the gravity acceleration, and ν is the velocity of the void relative to the resin matrix.

### 2.2. Mechanism of Uniform Heating by Microwave

#### 2.2.1. Correlation Model of Composite Materials during Microwave Heating

The microwave heating process involves the conversion of electromagnetic energy into thermal energy, with this energy being transmitted in the form of electromagnetic waves through space or media. The Poynting vector $\vec{S}\left(t\right)$ can be used to represent the electromagnetic energy passing through a unit area perpendicular to the direction of the electromagnetic wave propagation, representing the instantaneous electromagnetic power density. The Poynting vector can be expressed in complex form, as follows:

(8) E⇀t=Re[E⇀˙ejωt]=12[E⇀˙ejωt+E⇀∗˙e−jωt]

(9) H⇀t=Re[H⇀˙ejωt]=12[H⇀˙ejωt+H⇀∗˙e−jωt]

(10) S⇀t=E⇀t×H⇀t=14[E⇀˙×H⇀∗˙+E⇀∗˙×H⇀˙+E⇀˙×H⇀˙ej2ωt+E⇀∗˙×H⇀∗˙e−j2ωt]=12Re[E⇀˙×H⇀∗˙+E⇀˙×H⇀˙ej2ωt]

where $\dot{{\vec{E}}^{*}}$ and $\dot{{\vec{H}}^{*}}$ represent the conjugate forms of the instantaneous electric field intensity vector and the instantaneous magnetic field intensity vector. Integrating Equation (10) over a single period and calculating its average value, the following is obtained:

(11) Sav⇀=1T∫0TS⇀(t)dt=12Re[E⇀˙×H⇀∗˙]=Re[S⇀˙]

$\dot{\vec{S}}=\frac{1}{2}[\dot{\vec{E}}\times \dot{{\vec{H}}^{*}}]$ is referred to as the complex Poynting vector, representing the complex power density, where the real part represents the average power flux density, i.e., the active power density.

Furthermore, the power flux passing through a closed surface can be computed by integrating the complex form of the Poynting vector:

(12) ∮SP⇀⋅dS⇀=Re∮(E⇀×H⇀∗)2dS⇀

In the context of a time-harmonic electromagnetic field, Maxwell’s equations can also be expressed in complex form, as follows:

(13) $$\nabla \times \vec{H}=\vec{J}+j\omega \varepsilon_{0}\varepsilon^{*}\vec{E}$$

where $\varepsilon_{0}$ and $\varepsilon^{*}$ represent the vacuum permittivity and complex permittivity, respectively. Substituting $\vec{J}=\sigma \vec{E}$ and $\varepsilon^{*}=\varepsilon^{\prime }-j\varepsilon^{\prime \prime }$ into Equation (13) yields the following:

(14) $$\begin{array}{cc} \nabla \times \vec{H} & =\sigma \vec{E}+(\omega \varepsilon_{0}\varepsilon^{\prime \prime }+j\omega \varepsilon_{0}\varepsilon^{\prime })\vec{E}\\ & =\omega \varepsilon_{0}\varepsilon_{eff}\vec{E}+j\omega \varepsilon_{0}\varepsilon^{\prime }\vec{E} \end{array}$$

where $\varepsilon_{eff}=\varepsilon^{\prime \prime }+\sigma /\omega \varepsilon_{0}$ represents the effective loss factor, $\varepsilon^{\prime }$ is the dielectric constant, and $\varepsilon^{\prime \prime }$ denotes the loss factor, including all losses except conductivity. Taking the conjugate and multiplying both sides of Equation (14) by the electric field vector $\vec{E}$ yields the following:

(15) $$(\nabla \times {\vec{H}}^{*})\cdot \vec{E}=\omega \varepsilon_{0}\varepsilon_{eff}{\vec{E}}^{*}\cdot \vec{E}-j\omega \varepsilon_{0}\varepsilon^{\prime }{\vec{E}}^{*}\cdot \vec{E}$$

Similarly, rewriting Maxwell’s equation for the time-harmonic fields in complex form and multiplying both sides of the equation by the conjugate complex vector of the magnetic field intensity ${\vec{H}}^{*}$ results in the following:

(16) $$(\nabla \times \vec{E})\cdot {\vec{H}}^{*}=-j\omega \mu_{0}\mu^{\prime }\vec{H}\cdot {\vec{H}}^{*}$$

here, $\mu_{0}$ and $\mu^{\prime }$, respectively, represent the vacuum magnetic conductivity and the dielectric magnetic conductivity. Combining Equation (16) with Equation (15), the following is obtained:

(17) $$\begin{array}{cc} (\nabla \times \vec{E})\cdot {\vec{H}}^{*}-(\nabla \times {\vec{H}}^{*})\cdot \vec{E} & =-j\omega \mu_{0}\mu^{\prime }\vec{H}\cdot {\vec{H}}^{*}-\omega \varepsilon_{0}\varepsilon_{eff}{\vec{E}}^{*}\cdot \vec{E}\\ & +j\omega \varepsilon_{0}\varepsilon^{\prime }{\vec{E}}^{*}\cdot \vec{E} \end{array}$$

Integrating both sides of Equation (17) and applying the divergence theorem, the following is obtained:

(18) $$\begin{array}{cc} \oint_{V}\nabla \cdot (\vec{E}\times {\vec{H}}^{*})dV & =\oint_{S}(\vec{E}\times {\vec{H}}^{*})\cdot dS\\ & =-j\omega \oint_{V}\left(\mu_{0}\mu^{\prime }\vec{H}\cdot {\vec{H}}^{*}-\varepsilon_{0}\varepsilon^{\prime }\vec{E}\cdot {\vec{E}}^{*}\right)dV\\ & -\oint_{V}\omega \varepsilon_{0}\varepsilon_{eff}\vec{E}\cdot {\vec{E}}^{*}dV \end{array}$$

By integrating the differential form of the Poynting theorem over one period, averaging the result, and combining it with Equation (12), the expression for the average power of the dielectric loss can be obtained:

(19) Pav=−12∮SRe(E⇀×H⇀∗)⋅dS

Substituting Equation (18) into Equation (19), the following is obtained:

(20) $$P_{av}=\frac{1}{2}\omega \varepsilon_{0}\varepsilon_{eff}\oint_{V}(\vec{E}\cdot {\vec{E}}^{*})dV$$

Furthermore, when the electric field intensity vector is a constant, Equation (20) can be transformed into the following:

(21) $$P_{av}=\omega \varepsilon_{0}\varepsilon_{eff}E_{rms}^{2}V$$

where $E_{rms}$ represents the effective value of the electric field intensity, and V is the volume of the medium to be heated.

The heating rate of a composite material when subjected to an electromagnetic field and absorbing microwave energy can be calculated using Equation (22):

(22) $$P=\frac{Q_{t}}{t}=mc\cdot \left(\frac{T-T_{0}}{t}\right)$$

The expression for the heating rate of a composite material in the microwave field can be obtained by combining Equations (21) and (22):

(23) $$\frac{T-T_{0}}{t}=\frac{\omega \varepsilon_{0}\varepsilon_{eff}E_{rms}^{2}}{\rho c}$$

where ρ represents the density and c denotes the specific heat capacity of the composite material.

Equation (23) reveals that, in a microwave cavity with a consistent central frequency of irradiation, upon selecting the composite material, incident microwaves can be readily directed onto the surface and penetrate the material. The heating rate of the component is solely contingent on the square of the electric field strength inside the cavity, rendering it independent of the component’s size and structural intricacies. This effectively resolves the problem of non-uniform heating that is frequently encountered in traditional methods which employ hot air circulation for larger composite components with intricate features.

#### 2.2.2. Micro-Heating Mechanism of Composite Materials

**Figure 2:**
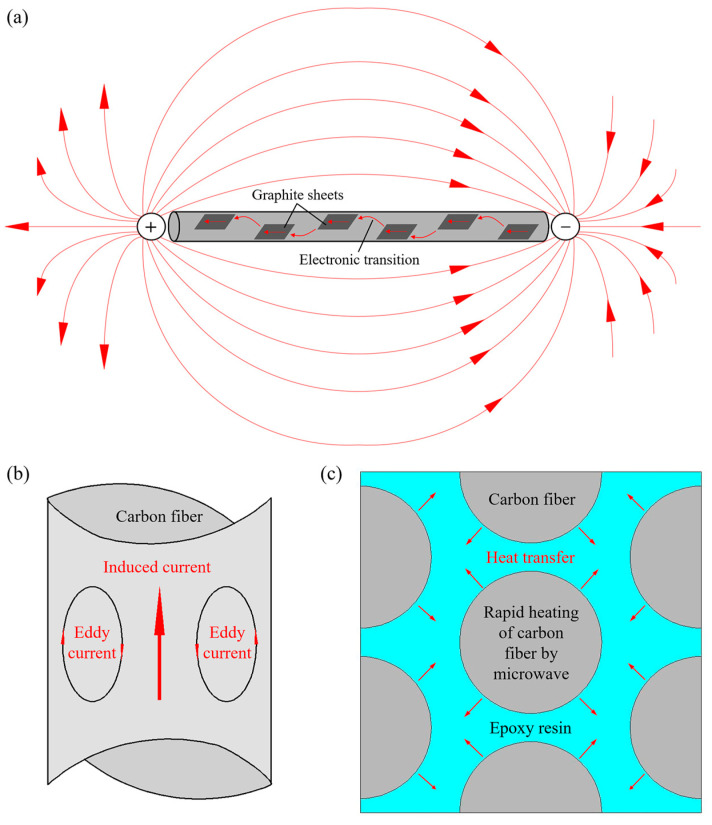
Microwave absorption performance of carbon fiber and epoxy resin: (**a**) resonance effect of carbon fiber in the electromagnetic field; (**b**) skin effect of carbon fiber caused by eddy currents; (**c**) diagram of heat transfer between fibers and resins.

During the microwave heating process of composite materials, the resonance effect played a significant role, wherein the carbon fiber acted as a harmonic oscillator when exposed to an alternating electromagnetic field. When the natural frequency of the carbon fiber matched the frequency of the electromagnetic waves, resonance occurred, resulting in the generation of induced currents inside and outside the fiber, as shown in Figure 2a.

The induced currents flowing within the fiber led to Joule heating due to the high contact resistance between the graphite sheets, effectively converting the microwave energy into thermal power. Additionally, these induced currents exhibited an uneven distribution across the fiber’s cross-section. As the microwave frequency increased, the variation in the internal currents intensified, leading to a greater concentration near the fiber’s surface, known as the skin effect, as illustrated in Figure 2b. The skin effect not only caused microwave energy attenuation via eddy current loss, but also introduced impedance loss on the surface of the fiber, effectively converting the microwave energy into thermal energy and heating the carbon fiber.

For epoxy resins containing polar groups, the polymer molecules exhibited dipole polarization, where their orientation aligned with the alternating electromagnetic field upon exposure to microwaves. Throughout this process, the movement of the polar molecules was impeded by inertial, elastic, and frictional forces between them, resulting in the conversion of microwave energy into internal molecular energy.

In summary, during the microwave curing process of composites, the superior microwave absorbing capability of carbon fibers compared to epoxy resins enabled the absorption of microwave energy by the fibers, which was subsequently converted into thermal energy and transferred to the surrounding resins through heat conduction, facilitating the crosslinking reaction and promoting the curing process, as shown in Figure 2c.

## 3. Materials and Methods

### 3.1. Materials and Equipment

**Figure 3:**
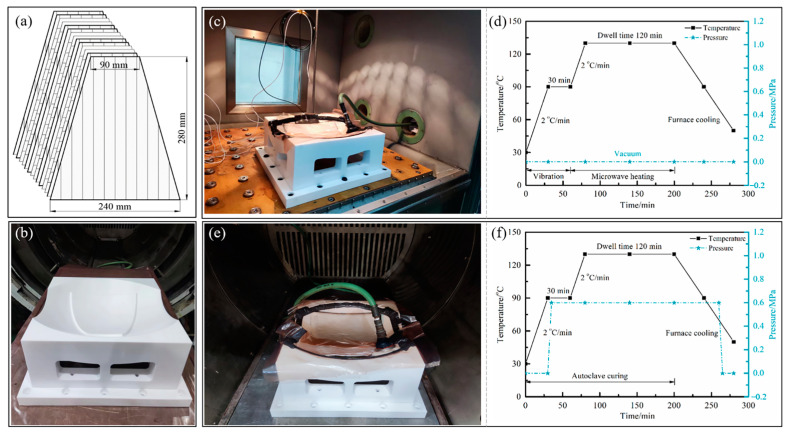
Manufacturing process of composite melon petal: (**a**) schematic of ply stacking; (**b**) PTFE mold; (**c**) vibration pretreatment; (**d**) curing curve of compound process; (**e**) autoclave process; (**f**) curing curve of autoclave process.

This study employed T800/#602 carbon fiber-reinforced composite materials, with a single-layer thickness of 0.17 mm, a density of 1.6 g/cm^3^, a fiber volume fraction of 65%, and a resin matrix of thermosetting epoxy, provided by the China Academy of Launch Vehicle Technology (CALT, Beijing, China). Following the ply stacking sequence of the composite tank prototype provided by the CALT, the melon petals were manually laid in a [0/0/90/90/0/0/90]s configuration. Based on the effective surface area calculation, the single prepreg layer was cut into a trapezoidal shape with dimensions of 90 mm on the top, 240 mm on the bottom, and a height of 280 mm, as shown in Figure 3a. The selection of the mold considered the need for the mold to have an absence of electromagnetic interactions and the ability to withstand vibration loads without deformation or damage. Therefore, in this study, a mold made of polytetrafluoroethylene (PTFE) with good microwave penetration was used as an alternative to traditional metal and glass molds, as illustrated in Figure 3b.

The vibration treatment equipment and microwave heating equipment used in the experiments were independently developed by the authors’ team. The vibration equipment had a limited working space of 0.9 m × 0.9 m × 1 m, with a vibrator frequency ranging from 10 to 5000 Hz and a temperature ranging from −70 to 200 °C. The platform was equipped with 13 air hammers capable of providing a vertical vibration acceleration of up to 70 g. To ensure that the vibrational excitation provided by the equipment effectively transmitted to the components, the components, after vacuum sealing, were securely fastened to the platform using bolts, as shown in Figure 3c.

The microwave heating equipment was configured with a cavity featuring a cross-section in the shape of a regular octagon. The cavity had an outer diameter of 1100 mm and a length of 1300 mm. Microwave generators were positioned along the length of each surface of the cavity, operating at a microwave frequency of 2.45 GHz. Each generator had a maximum output power of 1000 W, with the capacity for continuous adjustment within a range of 100–1000 W, and the cumulative maximum output power of the apparatus reached 8000 W. Furthermore, a mode stirrer constructed from 304 stainless steel was installed at the output port of each generator, facilitating the continuous reflection and superimposition of the microwave input by periodically rotating along the length of the cavity.

For the vibration pretreatment and microwave curing compound process, the melon petals were heated from room temperature to 90 °C at a rate of 2 °C/min and held at this temperature for 30 min while exposed to vibrational excitation with an acceleration of 10 g (where g = 9.8 m/s^2^). Following the completion of the vibration pretreatment, the melon petals were rapidly transferred to the microwave heating equipment. In the microwave heating stage, the components were heated from 90 °C to 130 °C and maintained at 130 °C for 120 min to facilitate the subsequent curing reaction, as depicted in Figure 3d. Throughout the compound process, only vacuum treatment was applied to the components.

To evaluate the compound process’s ability to reduce its reliance on pressure during the formation of complex curved components, while still meeting established standards for accuracy and quality, an autoclave process with the same temperature curve was employed as the experimental control group, but the curing pressure was consistently maintained at 0.6 MPa, as shown in Figure 3e,f, respectively.

### 3.2. Detection of Surface Profile Accuracy

A high-precision three-dimensional scanner, specifically the Infrared/Blue Laser Scanner (model: KSCAN-Magic, SCANTECH Co., Ltd., Hangzhou, China), was employed for the measurement of the surfaces of the melon petals produced via the two distinct processes. This equipment offers a maximum resolution of 0.01 mm and its highest accuracy level is 0.02 mm within the scanned area. The component surfaces underwent multiple scans, ensuring comprehensive coverage and confirming that there were no noticeable deficiencies in the component shapes. Subsequently, the test results were compared with the target model surfaces: using the target model surface as a reference, two endpoints at the larger end of the component and the midpoint at the smaller end were selected as reference points for comparison, and the relative deviations between the formed component surfaces and the target surfaces were finally obtained.

### 3.3. Microscopic Characterization

**Figure 4:**
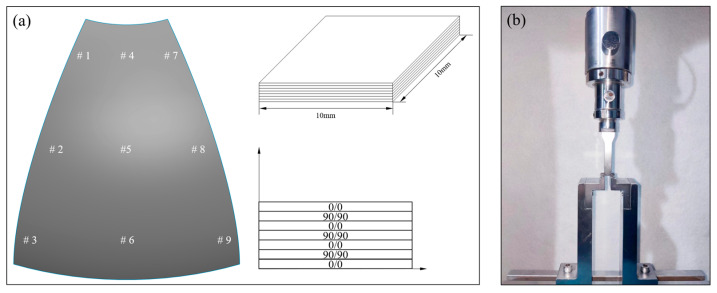
(**a**) Sample selection and dimensions for porosity analysis; (**b**) process of short-beam three-point bending test.

To understand the influence of different processes on the internal curing defects of the melon petals, a microscopic photography method was employed to analyze the void content and morphology within the components. Due to the complex geometric features of the curved petals, sampling was conducted in various regions of the components to better assess their overall porosity, as shown in Figure 4a. Samples for both the compound process and autoclave process were prepared in accordance with Chinese standard GB/T 3365-2008 [34], with dimensions of 10 mm × 10 mm. After preparation, sanding, polishing, and cleaning, the samples were examined using ODM (model: KETENCE VHX-5000) to analyze the microstructures of the internal defects. Image processing software (model: Image-Pro Plus 6.0) was employed to quantify the internal porosity of the components cured using different processes.

### 3.4. Mechanical Property Tests

The evaluation of interlaminar shear strength (ILSS) stands as a pivotal parameter in the assessment of interfacial characteristics within carbon fiber-reinforced composite materials. Short-beam three-point bending tests, known for their simplicity regarding equipment requirements and sample preparation, are widely favored for ILSS assessments in composite materials. For our analysis, samples were extracted from the designated regions of the melon petals marked in Figure 4a, and subsequent mechanical tests were conducted employing a universal testing machine (model: CMT-5105, MTS Systems Co., Ltd., Eden Prairie, MN, USA), as shown in Figure 4b. Since the tested samples, like the overall structure, had curvature, the dimensions of all samples were processed based on standard ASTM D2344/D2344M-16 [35]. The concave side of each sample was oriented downward, thereby situating the sample ends lower than the central point. The samples were aligned and centered, with the indenter and supports extending the sample width by 2 mm on each side; the indenter compression speed was set to 1 mm/min.

For a more comprehensive examination of the impact of both processes on the bonding properties of the fiber-matrix interfaces, SEM (model: EVO18, ZEISS Group, Oberkochen, Germany) was used to scrutinize the fracture surfaces of the tested specimens.

### 3.5. Cryogenic Permeability Tests

#### 3.5.1. Testing Principle and Equipment Construction

**Figure 5:**
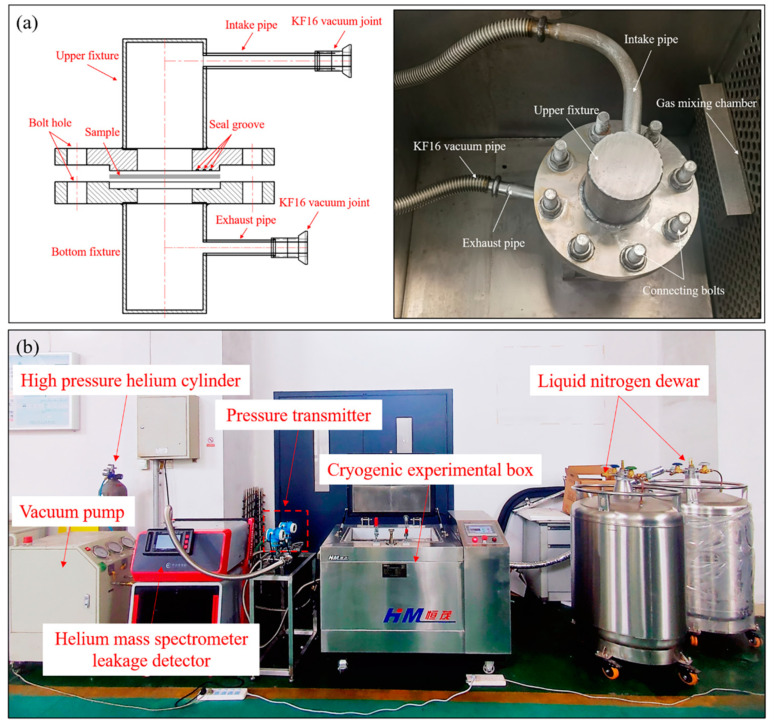
Cryogenic permeability tests of composite materials: (**a**) testing principle; (**b**) experimental device.

The permeation rate is commonly defined as the volume of gas that consistently passes through a specimen per unit time under constant temperature and pressure-differential conditions, with units expressed in $\mathrm{Pa}\cdot m^{3}/s$. In accordance with the Chinese standard GB/T 1038-2000 [36], this study involved the independent design and development of cryogenic permeability testing equipment specifically tailored to composite materials. The testing principle involves placing the specimen between two sealing fixtures, forming a sealed chamber with upper and lower sections, and thereby creating a high-pressure chamber and a low-pressure chamber, as shown in Figure 5a. Subsequently, high-pressure gas is introduced into the high-pressure chamber, while a vacuum pump evacuates the air from the low-pressure chamber to achieve a vacuum state. By using a pressure transducer connected to the low-pressure chamber, the incremental pressure within the low-pressure chamber per unit time can be recorded, enabling the calculation of the permeation rate of the specimen.

The cryogenic permeability testing apparatus for composite materials, as depicted in Figure 5b, primarily comprises a cryogenic experimental box (model: DY-W/0.08-CL, Xi’an HM Power Technology Co., Ltd., Xi’an, China) with external dimensions measuring 0.5 m × 0.4 m × 0.4 m. Both the inner and outer walls of the box are constructed from 304 stainless steel, and the interior is maintained at the set temperature via a 150 mm-thick polyurethane insulation layer. The system employs liquid nitrogen as a refrigerant and is equipped with two 200 L liquid nitrogen tanks to ensure extended operation at low temperatures. Liquid nitrogen is introduced into the box through a low-temperature solenoid valve and is adequately vaporized and cooled inside by a liquid nitrogen distributor and a circulation fan. Precise temperature control, ranging from ambient temperature to −190 °C, is achieved by regulating the evaporation of liquid nitrogen, with a temperature control accuracy of ≤±1 °C and temperature uniformity within the box of ≤±3 °C.

Considering the high precision required for equipment testing regarding the cryogenic permeability of composite materials, helium is chosen as the trace gas. A helium mass spectrometer leak detector (model: AT800, Shenzhen HESZK Technology Co., Ltd., Shenzhen, China) is connected to the low-pressure chamber and employed to detect gases that permeate from the high-pressure chamber through the sample into the low-pressure chamber. This detector offers an exceptionally high maximum detection precision, exceeding $1\times 10^{-13}\;\mathrm{Pa}\cdot m^{3}/s$, with a detection response time of less than 1 s. The leakage detection range spans from $1\times 10^{-13}\;\mathrm{Pa}\cdot m^{3}/s$ to $1\times 10^{-3}\;\mathrm{Pa}\cdot m^{3}/s$, and the detection port allows for a maximum pressure of 1500 Pa. The detector is equipped with a vacuum pump and a molecular pump, where the vacuum pump serves as the pre-stage pump for the pre-detection of components, and the molecular pump enables the detector to achieve a high vacuum state. A vacuum pipe is installed on the helium gas intake pipe, connected to a vacuum pump. This setup evacuates any residual air from the intake pipe and the high-pressure chamber before testing, effectively eliminating the influence of the helium gas present in the ambient air on the test results. Moreover, a high-precision pressure gauge is installed on the intake pipe, featuring a maximum range of 0.6 MPa and a minimum detectable pressure fluctuation of 10 Pa, which is used to precisely control the gas pressure injected into the high-pressure chamber. To maintain a sample temperature congruent with the environmental temperature set within the experimental chamber, all fixtures used for sealing the sample are placed inside the chamber. The composite samples are circular, with a diameter of Φ50 mm.

To ensure the accuracy of the test results and prevent inaccuracies stemming from suboptimal sealing within the testing system, all connecting pipelines employ high-vacuum flexible bellows. Furthermore, to fortify the connections between pipelines and joints, welding treatment is diligently applied. In consideration of the fact that rubber sealing rings, PTFE sealing rings, and similar materials tend to become brittle when exposed to cryogenic conditions, this study opts for the use of highly ductile indium for the preparation of sealing rings.

#### 3.5.2. Experimental Conditions

The prepared specimens are positioned between the high-pressure intake chamber and the low-pressure exhaust chamber, creating a secure seal via the utilization of indium sealing rings that are bolted together. To initiate the test, the ball valves of the vacuum pipeline, helium intake valve, and helium exhaust valve are opened. Simultaneously, the vacuum pump and the leak detector are activated, and the entire system’s pipeline undergoes a vacuuming process to eliminate any interference caused by helium contamination in the air. Upon the completion of the vacuuming process, the helium exhaust valve and the leak detector are left open, while the ball valves of the vacuum pipeline and the vacuum pump are securely closed. The controlled introduction of helium gas into the high-pressure chamber commences gradually through the helium intake valve, until the pressure gauge reads a consistent 0.5 MPa. Once this pressure is achieved, the helium intake valve is promptly closed, ensuring a sealed pathway within the system. The internal environmental temperatures of the cryogenic box are set to both 25 °C and −190 °C, respectively. After the initial stage of permeation rate variation over time, and the reading on the leak detector stabilizes, this reading is recorded as the cryogenic permeation rate of the specimen.

## 4. Results and Discussion

### 4.1. Analysis of Surface Accuracy

**Figure 6:**
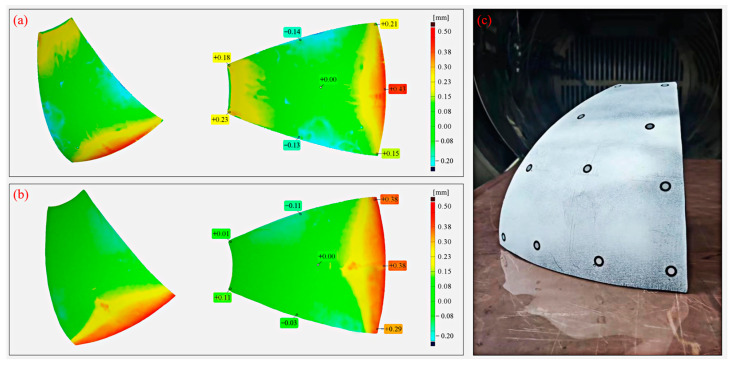
Deformation detection results of melon petals formed via different processes: (**a**) autoclave process; (**b**) compound process; (**c**) engineering prototype.

Figure 6a,b provides a visual representation of the analysis results, comparing the actual surfaces with the target surfaces of the components cured via both the autoclave and compound processes. It is evident that the deformation trends observed in the components produced by these processes are fundamentally aligned, with the most notable deviation occurring at the larger end of the component. This phenomenon can be attributed to the gradual increase in curvature near the larger end, leading to more pronounced deformation in this area during the actual forming process. For the autoclave-cured components, the maximum deviation is measured at 0.43 mm, while for those formed using the compound process, the maximum deviation is slightly less at 0.40 mm. The maximum deformations for both methods are quite similar.

Conversely, owing to the unique synchronous heating method of microwaves, the components fabricated using the compound process exhibit reduced deformation at the smaller end. The maximum deviation measures merely 0.11 mm, a significant improvement when compared to the 0.23 mm maximum deviation observed at the smaller end of the components manufactured using the autoclave process. It is noteworthy that both the autoclave process- and compound process-cured components display negative deformations of 0.16 mm and 0.11 mm, respectively, on their respective sides. These deviations are attributed to errors introduced during the actual layup and cutting stage of prepreg, and do not constitute deformations occurring during production.

The comparative analysis of the complex curved composite components produced via both processes revealed deviations within the range of ±0.6 mm. These findings not only align with the design requirements regarding surface accuracy in aerospace composite tanks, but also underscore the compound process’s ability to attain a comparable, or even superior, level of control over curing deformation in composite components when contrasted with the autoclave process.

### 4.2. Microstructure and Porosity Analysis

**Figure 7:**
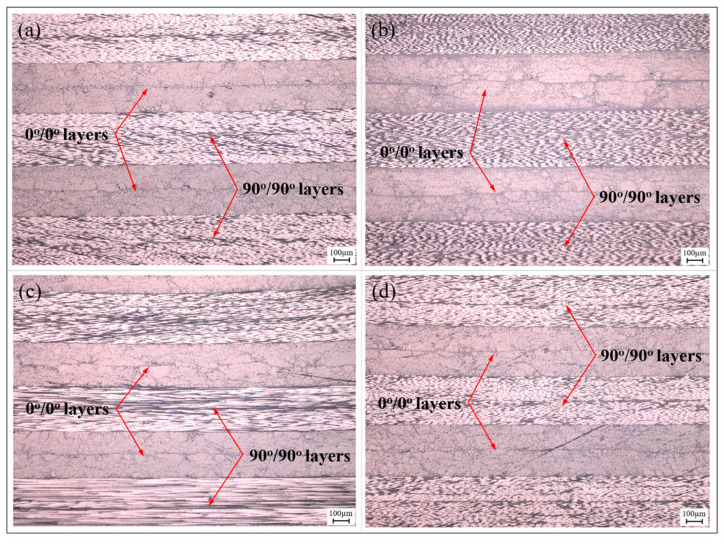
Microstructure of partial regions in autoclave-processed melon petal: (**a**) #2; (**b**) #4; (**c**) #6; (**d**) #8.

The internal microstructure of partial regions of the melon petals formed using the autoclave process is shown in Figure 7. It is evident that, across various curvatures of the component, the layers are closely combined, with the resin uniformly permeating the interstices between the fiber bundles. No noticeable voids or delamination defects are observed within or between layers, both along the carbon fiber direction and perpendicular to it. Given the curing pressure of 0.6 MPa, the static hydrostatic pressure induced by the resin matrix surpasses the pressure within voids. This action serves to suppress void expansion and coalescence, while potentially prompting voids to undergo collapse in response to high-pressure conditions, allowing them to dissolve back into the resin matrix. Furthermore, the pressure differential along the thickness of the component progressively encourages resin infiltration between fiber bundles as the curing temperature increases, further enhancing the resin’s wettability and bonding properties while eliminating the voids present between fiber bundles.

**Figure 8:**
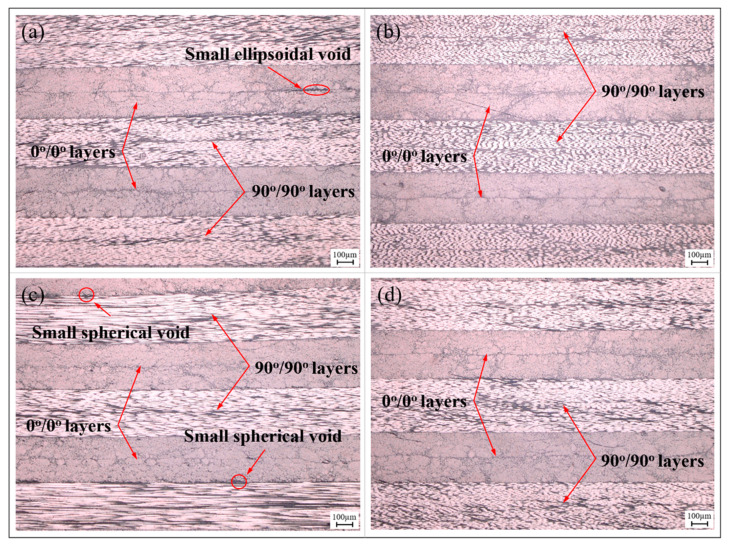
Microstructure of partial regions in compound-processed melon petal: (**a**) #2; (**b**) #4; (**c**) #6; (**d**) #8.

**Figure 9:**
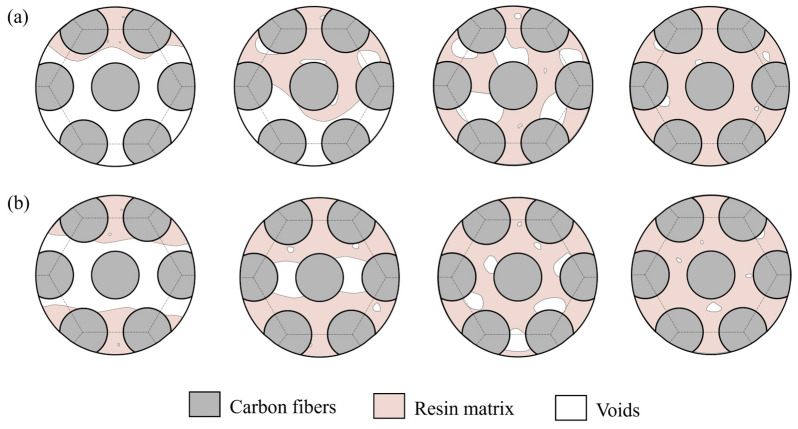
Illustration of resin infiltration and void formation: (**a**) autoclave process; (**b**) compound process.

Figure 8 illustrates the internal microstructure of the components produced using the vibration pretreatment and microwave curing compound process. Similar to the components manufactured through the autoclave process, no significant voids or delamination defects are observed in cross-sections parallel and perpendicular to the fibers. Only a small number of small-sized voids exist at the interfaces between layers in specific areas of the component, primarily taking on circular and elliptical shapes. This phenomenon strongly indicates that the reduction and inhibition effects of vibration pretreatment on curing defects in composite materials are not limited to structurally simple laminates, but are equally applicable to components with complex structural features and variable curvatures. Differing from the autoclave process in which resins infiltrate carbon fibers layer by layer and eliminate voids via high pressure, under the excitation of vibration energy, voids exhibit significant dynamic response characteristics; this not only promotes a reduction in their interface area and volume, but also accelerates their contraction due to surface tension. Simultaneously, the input of vibration energy also positively affects the resin’s flowability, contributing to the buoyant movement of voids within the components, as shown in Figure 9. Ultimately, this leads to a reduction in and the suppression of curing defects in the form of void collapse and escape.

Further statistical comparisons were conducted on the internal porosity within various regions of the melon petals formed using both processes. As the melon petals maintain a uniform thickness, the autoclave’s high curing pressure can be evenly distributed across a component’s surface, resulting in consistent defect inhibition across different regions. Consequently, the statistical porosity data for the autoclave-cured melon petals show overall consistency, with an average porosity of 0.28%. In contrast, the components manufactured via the compound process exhibit slight variations in their porosity among different sampling areas. This can be attributed to enhanced resin flow within the component as the curing temperature rises. The elevated temperature prompts the flow to tend toward the central region with a larger curvature without external pressure, influencing the void escape patterns and the resin’s infiltration behavior at the edge regions. Consequently, higher porosity is observed in these areas. Nevertheless, this variation is not substantial, with the statistical data indicating a mere 0.08% difference between the regions with the highest and lowest internal porosities.

The average porosity within melon petals produced via the compound process measures 0.39%, demonstrating that the compound process yields a defect inhibition effect akin to that of the 0.6 MPa autoclave process, even for complex curved components.

### 4.3. Analysis of Interlaminar Bonding Property

**Figure 10:**
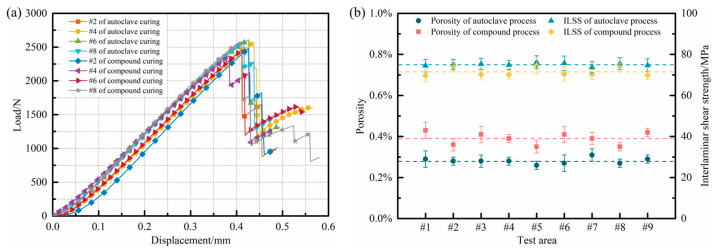
(**a**) Typical load–displacement curves; (**b**) comparison of internal porosity and ILSS in melon petals formed via different processes.

The interfacial properties between the fibers and resins were assessed using short-beam three-point bending tests, and the results obtained from these tests are presented in Figure 10.

Figure 10a depicts the representative load–displacement curves of the samples produced via the distinct processes. Generally, the curves exhibit similar trends as well as the maximum loads, and can be divided into multiple stages. In the initial stage, the load increased linearly as the displacement grew until reaching the maximum load. Subsequently, in the second stage, cracks developed at the sample’s interface, resulting in an instantaneous drop in its load-bearing capacity. However, due to incomplete debonding between the sample layers, the cracks continued to propagate, leading to a rebound in the curve as the loading displacement increased during the third stage. Eventually, as the cracks reached the limit state, the interfaces of the sample underwent complete debonding and destruction, causing a sharp decline in the curve again.

Moreover, the ILSS of each sample was calculated, as illustrated in Figure 10b. It is evident that the ILSS of melon petals fabricated via the 0.6 MPa autoclave process consistently surpasses 73 MPa at various positions, indicating exceptional interfacial quality. The application of a high curing pressure effectively eradicates nearly all internal defects within the components. The resin progressively infiltrates the fiber bundles layer by layer and comprehensively wets them throughout the flow process, leading to a noteworthy enhancement in the interfacial bonding performance.

The melon petals produced via the compound process under low-pressure conditions exhibit ILSS values in various regions that are comparable to those of the components formed via the autoclave process, with an average ILSS of 71.51 MPa. The introduction of vibration pretreatment not only effectively reduces and suppresses the generation of internal defects such as voids and delamination within the components, but also induces periodic resin movement within the structures. This enhanced flowability of the resin under low-pressure conditions facilitates more thorough penetration between the fiber bundles. Additionally, influenced by the preferential heating of carbon fibers within the microwave field, the cross-linking reactions occurring on the surfaces of the fiber bundles become more complete, leading to a further increase in the bonding strength between the resin and the fibers.

**Figure 11:**
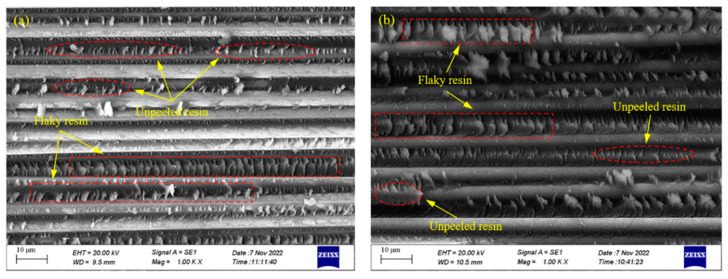
SEM micrographs of samples after mechanical tests: (**a**) autoclave process; (**b**) compound process.

A further examination of the horizontal fracture surfaces of the specimens following three-point bending tests was performed using SEM, as illustrated in Figure 11. In the samples cured via the autoclave process, the resin effectively infiltrated the interstices between the fiber bundles due to the application of a high curing pressure, allowing the fiber bundles to offer ample support while bearing loads. Moreover, the high curing pressure greatly promoted resin impregnation and the encapsulation of the carbon fibers, resulting in a well-established interfacial bonding state within the components.

Specimens formed via the compound curing process exhibited similar morphological features on their horizontal cross-sections. The introduction of the vibration energy field enabled the resin to flow freely between, and fill the gaps between, the fiber bundles, preventing the excessive accumulation of resin between them. This facilitated the effective transfer of external loads to the carbon fibers, generating uniformly distributed flaky resin between the fiber bundles under shear forces. Additionally, the preferential heating effect of carbon fibers in the microwave field promoted the complete resin impregnation of the surrounding areas, leading to the even envelopment of carbon fiber surfaces by the resin matrix. The above actions enhanced the adhesive strength between the resin and fibers, fully utilizing the outstanding mechanical properties of carbon fibers. Consequently, following the shear failure of the specimens, a substantial amount of adhered, unpeeled resin residue was observed on the carbon fiber surfaces.

### 4.4. Analysis of Ambient/Low Temperature Permeability

#### 4.4.1. Validation of Equipment Sealing Performance

To verify the sealing performance of the testing device in low-temperature and pressure-differential conditions, and to ensure the accuracy and reliability of the subsequent experimental results, sealing performance tests were conducted under both ambient and low-temperature environments. The specimens were prepared using 2219 aluminum–copper alloy sheets, known for their excellent anti-leakage properties. Each specimen had a thickness of 2 mm and a diameter of Φ50 mm. They were meticulously clamped and sealed using fixtures to prevent any potential permeation from influencing the device’s sealing performance test outcomes.

**Figure 12:**
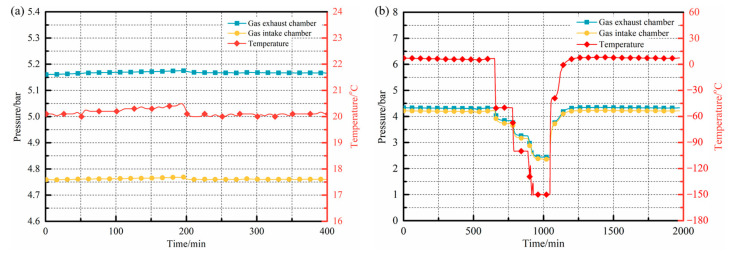
Sealing performance of equipment: (**a**) ambient temperature condition; (**b**) cryogenic condition.

For the sealing performance tests conducted at ambient temperature conditions, the device was maintained at a constant temperature of 20 °C. Helium gas was introduced into the high-pressure and low-pressure chambers separately, at pressures of 0.52 MPa and 0.48 MPa, via dedicated intake and exhaust pipes. Following pressurization, all ports were sealed, and the pressure variations within the chambers were monitored and recorded using high-precision pressure gauges installed in the intake and exhaust pipes, as illustrated in Figure 12a. As depicted in the figure, when the environmental temperature within the experimental box remained stable, there was negligible gas pressure fluctuation observed within the two sealed chambers over time. After 200 min, a notable reduction in the environmental temperature led to synchronous changes in the gas pressure within both chambers, following a consistent trend in line with the expectations dictated by the ideal gas law.

Furthermore, by pre-setting a stepwise cooling curve within the system, the precise temperature control capacity of the equipment and its sealing performance across a range of low-temperature conditions were further validated. The temperature range of the tests covered from room temperature to −150 °C, and the results are shown in Figure 12b. As depicted in the figure, the pressure within both the upper and bottom sealed chambers exhibited synchronous changes in response to variations in the environmental temperature under these low-temperature conditions. Moreover, when the environmental temperature remained constant, the current pressure was maintained, reflecting the excellent sealing performance of the device under different low-temperature environments. Additionally, a comparison of the pressure values within the two sealed chambers before and after the experiments showed that, prior to cooling, the pressure in the upper intake chamber and bottom exhaust chamber was 4.216 bar and 4.334 bar, respectively. Following the conclusion of the test and the return to room temperature, the corresponding pressures within the two sealed chambers were 4.216 bar and 4.330 bar, respectively. Throughout the entire testing procedure, the pressure within the sealed chambers remained constant, providing evidence of an effective seal without noticeable gas permeation within the system.

In summary, the permeability testing equipment developed in this study exhibited an exceptional sealing performance under various conditions, including both ambient and low-temperature environments, which served as a valuable resource for conducting further experiments.

#### 4.4.2. Permeation Rates of Components

**Figure 13:**
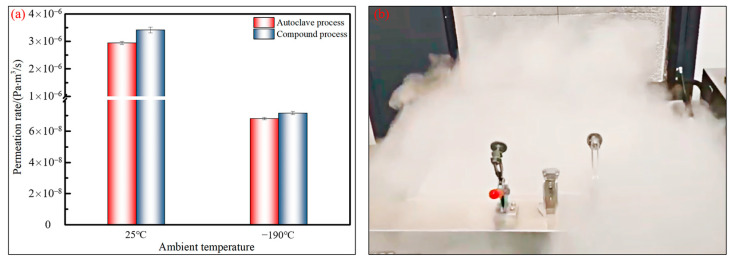
Permeation tests of composite melon petals: (**a**) test results; (**b**) cryogenic testing process.

Figure 13 presents the helium permeation rates of the composite melon petals cured using two different processes under test conditions at both room temperature (25 °C) and cryogenic temperature (−190 °C). The figure reveals that the permeation rates of the melon petals prepared via the autoclave process and the compound curing process exhibit consistency at both temperature conditions. At 25 °C, the permeation rate for melon petals formed via the autoclave process reaches $2.94\times 10^{-6}\;\mathrm{Pa}\cdot m^{3}/s$, while for components formed via the compound process, it remains within the same order of magnitude, specifically $3.41\times 10^{-6}\;\mathrm{Pa}\cdot m^{3}/s$. Combining the above defect statistics, it becomes apparent that the internal defect morphologies of the components produced via the two processes are generally similar, with porosities falling within a relatively low range. Small-sized voids, dispersed irregularly within the components, do not form nearly continuous permeation channels along the thickness direction of the specimens. Consequently, the limited number of curing defects minimally impacts the permeation pathway and the behavior of small-molecule cryogenic media within the components. This fundamental fact explains why there is no significant difference in the permeation rates between the components fabricated via the two processes.

As the experimental environment reached a low temperature of −190 °C, the permeation rates of the components fabricated using the different processes experienced a substantial reduction, transitioning from the order of $10^{-6}\;\mathrm{Pa}\cdot m^{3}/s$ to the order of $10^{-8}\;\mathrm{Pa}\cdot m^{3}/s$. This phenomenon is attributed to the fact that, within composite components characterized by a low number of defects and the absence of pronounced damage, the gas employed in testing primarily undergoes permeation along the thickness direction via molecular diffusion. This behavior can be well-described by the generalized chemical control Equation (24):

(24) $$J=-SD\cdot \left[\frac{\partial \phi }{\partial x}+\kappa_{S}\frac{\partial }{\partial x}\left(\ln\left(\theta -\theta_{Z}\right)\right)+\kappa_{P}\frac{\partial P}{\partial x}\right]$$

where J represents the concentration flux of the diffusing gas; S stands for the solubility of the diffusing gas; D represents the diffusion coefficient; C is the concentration of the diffusing gas; ϕ=C/S represents the normalized concentration; $\kappa_{S}$ denotes the Soret effect factor, which controls the temperature-driven diffusion behavior; $\kappa_{P}$ is the pressure stress factor, which governs the pressure-driven diffusion behavior; θ is the environment temperature; $\theta_{Z}$ represents absolute zero temperature; and P is the equivalent pressure. In practical testing scenarios, where temperature-driven gas diffusion effects can be ignored, the generalized chemical control equation can be further simplified to an extended version of Fick’s law:

(25) $$J=-D\left(\frac{\partial C}{\partial x}+S\kappa_{P}\frac{\partial P}{\partial x}\right)$$

According to Equation (25), it becomes evident that a decrease in the environmental temperature corresponds to a decrease in the average kinetic energy of gas molecules, leading to reduced molecular thermal motion. Consequently, the diffusion coefficient D gradually decreases, resulting in a declining concentration flux of the diffusing gas. Simultaneously, in accordance with the ideal gas state equation, the reduction in ambient temperature leads to a decrease in gas pressure within the high-pressure chamber. Consequently, the effective pressure P acting on the specimen differs from that experienced under ambient temperature conditions. The combined effect of these factors is macroscopically reflected as a decrease in the cryogenic permeation rate.

A further comparison of the permeation rates at −190 °C for melon petals fabricated using different processes reveals that the test result for the melon petals manufactured using the autoclave process is $6.81\times 10^{-8}\;\mathrm{Pa}\cdot m^{3}/s$, while that for the petals manufactured using the compound process is $7.17\times 10^{-8}\;\mathrm{Pa}\cdot m^{3}/s$. These cryogenic gas permeation rates for both types of components are within the same order of magnitude, indicating no significant difference. Therefore, by introducing vibration pretreatment into the composite microwave heating and effectively reducing the amount of defects during the curing process, a lower defect content, smaller void sizes, and a more discrete distribution pattern were achieved. This prevented the formation of permeation channels for small-molecule media within the composite melon petals at low temperatures, enhanced the adsorption capacity of the resin matrix, and, under low-pressure curing conditions, resulted in permeation rates comparable to those of components manufactured using the standard 0.6 MPa autoclave process.

## 5. Conclusions

In this study, aiming to address the challenges of achieving the integrated and cost-effective manufacturing of aerospace cryogenic composite tanks that cannot be realized through the conventional autoclave process, and of those in existing out-of-autoclave processes which are unable to effectively suppress defects under low-pressure conditions, a vibration pretreatment was innovatively introduced into the microwave curing process of composite materials. Based on a systematic analysis of the inhibitory mechanisms of vibration pretreatment on void formation and the uniform heating mechanisms of microwaves in composite materials, the main conclusions are as follows:

(1) The composite melon petals formed via the vibration pretreatment–microwave curing compound process exhibited excellent shaping precision, with the maximum deformation occurring at the larger end of the components, being approximately 0.40 mm. This deformation was less than the ±0.6 mm surface precision requirements for engineering applications;
(2) There were no large-sized voids or delamination defects at various positions within the components, and the interior average porosity was only 0.39%, which demonstrated that the compound process was equally effective in reducing and inhibiting voids in the manufacturing of composite components with complex structural features;
(3) The compound process fully leveraged the advantages of vibration pretreatment in inhibiting the generation of defects and microwave curing in enhancing the interfacial bonding performance, and the average ILSS of the cured melon petals reached 71.51 MPa. A sufficient fiber impregnation, together with a strong interfacial adhesion between the fibers and the matrix during the curing process, was obtained, thus increasing the capacity for load transfer from the matrix to the fibers through the interfaces.
(4) At a pressure differential of 0.5 MPa, the melon petals cured via the compound process exhibited permeation rates of $7.17\times 10^{-8}\;\mathrm{Pa}\cdot m^{3}/s$ and $3.41\times 10^{-6}\;\mathrm{Pa}\cdot m^{3}/s$ at room temperature and low temperature, respectively. These results were within the same order of magnitude as those of the components manufactured using the standard autoclave process, thereby meeting the requirements for aerospace cryogenic composite tanks in resisting the permeation of small-molecule media.

The integration of vibration pretreatment into the microwave curing process enabled the production of components under low-pressure conditions, while achieving surface precision and comprehensive properties that were equivalent to those obtained via the standard 0.6 MPa autoclave process. This substantial reduction in the reliance on curing pressure represents a significant advancement in the fabrication of high-performance composite materials and holds promising engineering applications for out-of-autoclave processes in the manufacturing of large-scale aerospace cryogenic composite tanks.

## Data Availability

The data presented in this study are available upon request from the corresponding author.
